# A survey on the influence of CYBATHLON on the development and acceptance of advanced assistive technologies

**DOI:** 10.1186/s12984-022-01015-5

**Published:** 2022-04-02

**Authors:** Jan T. Meyer, Selina Weber, Lukas Jäger, Roland Sigrist, Roger Gassert, Olivier Lambercy

**Affiliations:** 1 Rehabilitation Engineering Laboratory, ETH Zürich, Zürich, Switzerland; 2 CYBATHLON, ETH Zürich, Zürich, Switzerland; 3 Future Health Technologies, Singapore-ETH Centre, Campus for Research Excellence And Technological Enterprise (CREATE), Singapore, Singapore

**Keywords:** CYBATHLON, Advanced assistive technology, User-centered design, Exoskeleton, Prosthesis, Wheelchair, Functional electrical stimulation

## Abstract

**Background:**

Advanced assistive technologies (AAT) aim to exploit the vast potential of technological developments made in the past decades to improve the quality of life of people with disabilities. Combining complex robotic technologies with the unique needs of people with disabilities requires a strong focus on user-centered design to ensure that the AAT appropriately addresses the daily life struggles of target users. The CYBATHLON aims to promote this mindset by empowering the AAT target users (“pilots”) to compete on race tracks that represent approximations of daily life obstacles. The objective of this work was to investigate the AAT technology development, usability, and user involvement (i.e., application of user-centered design) in the context of the CYBATHLON.

**Methods:**

With an online survey targeting the pilots and technical leads of teams preparing for the CYBATHLON 2020 Global Edition, we investigated to what extent the pilots were involved in device development and how this influences the perceived usability of the showcased AAT. Furthermore, the effects of user-centered design variables on the individual race performances were analyzed.

**Results:**

A total of 81 responses from 35 pilots and 46 technical leads were collected in the two weeks prior to the event. Of all teams partaking in the included disciplines of the CYBATHLON 2020 Global Edition, 81.8% (36 of 44) were included in the study. User-centered design appeared to be a prevalent practice among the teams, as 85.7% of all pilots reported a certain level of involvement. However, only 25.5% of the pilots reported daily life usage, despite QUEST usability scores of both respondent groups showing moderate to high satisfaction with the respected AAT across all investigated disciplines. An explorative linear mixed model indicated that daily life usage (p < 0.05) and prolonged user involvement (e.g., more than 2 years, p < 0.001) have a significant positive effect on the race performance at the competition.

**Conclusions:**

We conclude that the CYBATHLON positively fulfills its conceptual goals of promoting active participation and inclusion of people with disabilities in the design and evaluation of AAT, thereby stimulating the development of promising novel technological solutions. Also, our data could underline the value of the competition as a benchmark, highlighting remaining usability limitations or technology adoption hurdles.

**Supplementary Information:**

The online version contains supplementary material available at 10.1186/s12984-022-01015-5.

## Background

Assistive technologies such as wheelchairs, prosthetics, and orthotics can be used to restore or replace lost functionalities and activities for the daily life of people with disabilities [1]. Technological advancements within the last two decades allowed integrating more functions into such devices, bringing forward advanced assistive technologies (AAT), such as wearable robots to tackle remaining daily life hurdles. Unfortunately, such AAT are not only more complex and costly, but likely require technical support to be used. Current AAT solutions therefore seldom satisfy the target user in several decisive usability aspects. Consequentially, thousands of AAT are either abandoned soon after acquisitions or may not get accepted in the first place due to their limited usability in daily life [2, 3]. A potential solution to tackle the prevalent acceptance issues of AAT is the active involvement of technology stakeholders in device development [4–7]. Numerous guidelines for such user-centered design (UCD) of AAT have evolved of the past two decades, proposing similar approaches to promote the consideration of complex user needs in human–robot interactions [8–11]. Unfortunately, the early and active involvement of target users with special needs is easier said than done. User-centered design not only requires substantial resources, but most importantly the willingness, openness, and availability of people with disabilities and technology makers to form such interdisciplinary development teams.

The CYBATHLON, organized by ETH Zurich, Switzerland, is a unique championship tackling this issue by promoting AAT usability and technology acceptance in a competitional format [12]. Within six different race disciplines, people with physical disabilities, also called “pilots,” compete by completing everyday tasks and racetracks using state-of-the-art AAT. Besides its aims to promote inclusion and to remove barriers for a more accessible world, one of the main goals of CYBATHLON is to “*promote research, development, and implementation of assistive technology for people with disabilities”* [12–14]*.* Through the nature of the event, the pilots of the AAT become an active part of the teams participating, as their skills in handling the device influence how successful the obstacle courses and races can be completed. Therefore, it is expected that the CYBATHLON actively encourages UCD to promote device usability and technology acceptance [13, 14]. Moreover, the CYBATHLON transitioned from being a sheer competition, to become a platform that may contribute to AAT evaluation and development benchmarking [15–18]. The influence and effects of the CYBATHLON on the AAT field and specific technologies have so far only been shown in discipline-specific summaries or individual success stories [20–27]. A structured, multi-disciplinary analysis of the platform’s influence on user involvement and AAT usability and conclusions from the teams’ performances at the competition are yet missing.

In order to answer some of these questions, we aimed to take advantage of the CYBATHLON 2020 Global Edition to collect data from an exclusive pool of international teams, their users, and AAT devices, to get a unique understanding of their development practices and user experiences. More specifically, we wanted to understand (1) if the CYBATHLON does in fact promote active involvement and consideration during device development, (2) if the devices showcased at the CYBATHLON are also usable in daily life, and (3) if the user involvement, device usability, and usage intensity are indicators of performance when it comes to the CYBATHLON competition.

In this work, we present the results from a multi-lingual, online survey administered to the users (pilots) and developers (technical leads) of the teams at the CYBATHLON 2020 Global Edition. A total of 81 responses from 44 teams were analyzed to understand the platform’s influence on AAT development strategies in terms of user involvement, perceived usability, and performance indicators. We discuss how the CYBATHLON inherently promotes user-centered design and the effect of user involvement on device usability and race performances.

## Methods

### Study design

The device development for participation at the CYBATHLON is driven by two main user groups: the technical leads (TL) and the team pilots (PIL). Therefore, an online survey for the PIL and TL of teams participating at the CYBATHLON 2020 competition was designed. The survey consisted of shared (both groups) and specific (one group) questions for the respective respondent groups, with a total of 26 questions for the PIL and 30 for the TL. The survey was administered using the QuestionPro Survey Software (QuestionPro Inc., Austin, TX, USA). Standardized and validated question formats such as Likert scales and multiple-choice questions were used, and survey-dry runs with several independent reviewers was performed to eliminate potentially misleading, overcomplicated, or double-barreled questions. All survey questions and research aims were discussed and approved among the authors, assuring that face validity was established. The survey contained initial questions on demographics, then covered the respondent involvement and role in device development, followed by assessments of usability and daily life applicability of the CYBATHLON specific AAT. Furthermore, the survey investigated topics such as the importance and intensity of task-specific training or the user interfaces and control strategies used and preferred by the PIL. In this work, only a selection of questions relevant to the central message of this paper will be presented in detail. The full survey is accessible in the supplementary materials. As not all TL and PIL understand English, both surveys were provided in nine additional languages: Brazilian Portuguese, French, German, Greek, Italian, Japanese, Korean, Polish and Russian. The different survey languages were prepared and provided based on the demand of the participating international teams. The original English version was translated using the DeepL translation service (DeepL LLE, Cologne, Germany), or by professional translators where not available (Korean, Japanese). Each of these translations was additionally validated by a native-speaking expert familiar to the rehabilitation engineering field. On the online survey landing page, the study aims were presented, and informed consent was collected form the participants. Once the participants agreed with the stated terms and conditions, the surveys started.

### Duration, intensity, and quality of user involvement

From previously shared stories and anecdotes from teams that participated in the CYBATHLON, we understand that it is common practice among the teams for the PIL to be the center of attention. They are the ones representing the teams on an international stage, as faces of universities, companies, individual makers, and sponsors. The overall concept of the CYBATHLON therefore already implies that a certain level of user-centeredness is necessary within the device development and preparation to the competition in order to be successful. To understand if and how UCD is applied among the CYBATHLON teams, we asked questions on the duration, intensity, and quality of user involvement to both the PIL and TL. For example, the duration of involvement was rated in months and years, with the following ordinal intervals provided: *No, not at all, Less than 6 months, 6–12 months, 1–2 years, 2–4 years, More than 4 years*. Respondents who selected “*No, not at all*” were asked to specify why they/the user were not involved in the development at all. A specific form of user involvement is the actual usage, i.e., training with the device. Therefore, the PIL were asked to specify the hours of training per week (intensity) and total time of training in months/years (duration) which they spent with the device, preparing for the CYBATLON competition. Assuming user-centered design, we asked the respondents to rate their level of involvement from 0 = *not involved at all*, to 100 = *leading role*, within each of five distinct development phases: (1) empathize, (2) define, (3) ideate, (4) prototype, and (5) evaluate. The phases were adapted from universal Design Thinking methodologies [28]. PIL with a leading role across the whole development process could get a maximum involvement score of 500 (5 × 100). Further, both the TL and PIL were asked to rate their level of agreement (5-point Likert scale) with three statements on PIL involvement during the device development.

### Device usability and daily life applicability

Usability, as defined by the International Organization for Standardization (ISO) as “extent to which a system, product or service can be used by specified users to achieve specified goals with effectiveness, efficiency, and satisfaction in a specified context of use”, plays a significant role in the technology transfer and acceptance of AAT [8]. Most usability studies with AAT consist of only few trials, or even single sessions, in which the user gets to try a device and is asked to rate the usability afterward [29]. Assessing the usability from the perspectives of PIL and TL who have been actively using and developing the devices for a more extended period of time, therefore, might generate a seldom reported, long-term use view of AAT usability. A summative usability evaluation was performed using the device subscale of the Quebec User Evaluation of Satisfaction with Assistive Technology (QUEST 2.0) [30]. The standardized, 5-point Likert scale individually assesses the user's satisfaction with the following device attributes: *dimensions*, *weight*, *adjustment*, *durability*, *comfort*, *ease of use*, *effectiveness*, and *safety*. The reported QUEST score is the overall mean of all individual attributes and ranges from 1 = not satisfied at all to 5 = completely satisfied. Another more meaningful measure of device usability is the actual daily use. For that reason, we specifically asked the PIL if, for which activities, and for how long they have used their device in everyday life outside of CYBATHLON usage. Respondents who indicated not to use their device in daily life were asked to specify the most limiting factors restricting the daily use.

### Sample

At the CYBATHLON competitions, a global selection ranging from student teams to established AAT companies compete in six different disciplines: 1. Powered arm prosthesis (ARM), 2. powered leg prosthesis (LEG), 3. powered exoskeleton (EXO), 4. powered wheelchair (WHL), 5. functional electrical stimulation (FES), and 5. brain-computer interface (BCI) [12]. While the races of disciplines ARM, LEG, EXO, and WHL are obstacle courses with tasks resembling activities of daily living, FES and BCI teams compete upon the longest distance covered during a given timeframe. As a consequence of the worldwide travel and interaction restrictions during the COVID-19 pandemic, the format of the CYBATHLON 2020 was changed to a decentralized, "Global Edition." Teams participating from foreign countries organized their own hubs by rebuilding the obstacle course and live-streamed their races to the organizers and referees in Switzerland. The individual race recordings took place within the week prior to the live-streamed event. This study considered TL and PIL of all teams participating in disciplines ARM, LEG, WHL, EXO, and FES of the CYBATHLON 2020 Global Edition. Teams of the BCI discipline were not included as they were intended to participate in another, independent, discipline-specific study at that time. Data were collected in the two weeks prior to the global streaming of the event on November 13–14th, 2020. All teams were contacted via e-mail and invited to an individual video conferencing call with at least one survey coordinator in order (1) to assist with the access to, and completion of the online survey, and (2) to make sure the PIL and TL complete their surveys independently. Teams unable to make time for a call were provided with the survey link via e-mail and clear instructions on how to complete the survey in order to avoid further bias of the respondents completing the surveys.

### Data analysis

Each team was provided with a randomly generated, four-digit team-ID, which enabled the study coordinators to perform intra-team data analyses. All written answers (e.g., when specifying “other”) were translated to English for data analysis. The raw survey data was exported from QuestionPro as EXCEL reports. Further post-processing and the statistical analysis of the survey data were done in MATLAB R2021a (MathWorks, MA, USA) and RStudio Team 2021 (RStudio PBC, MA, USA). Two-sample t-tests (*ttest2*, MATLAB), were applied at a 0.05 significance level under equal variance assumptions to investigate the QUEST scores between disciplines or between PIL and TL data samples. As an explorative analysis, a linear mixed-effects model was established to investigate PIL variables influencing the performance (relative rank) at the competition. As the CYBATHLON competition scoring system differs between all categories, and is a combination of the individual task success and task time, we derived a normalized, dependent variable called “relative rank” (RR) based on the ranking of the teams within their discipline at the CYBATHLON 2020 Global Edition [Source: www.cybathlon.ethz.ch]:

$$RR= 100 - ( \frac{Final\ rank\ in\ discpiline-1}{ Number\ of\ teams\ in\ dscipline - 1 } *100)$$

A RR of 100 indicates that the team ranked first in their discipline, and RR = 0 was given to teams that came in last. Only PIL variables were considered for the mixed-effects model, as the RR mainly relies on their performance and experience. The following variables were included in the model: QUEST_score (continuous, 1–5), daily_use (categorical, 2 levels), involvement_quality (continuous, 0–500), involvement_duration (categorical, 5 levels), training_intensity (categorical, 4 levels), training_duration (categorical, 4 levels). Discipline (categorical, 5 DOF) was added as a random effect since the RR are grouped by discipline. Rating the usability of a device depends on the context of use and perspective for which the user rates a system. We thus assumed that the QUEST score is dependent on daily use, as device usage in daily life differs significantly from CYBATHLON specific training only. An interaction effect was thus investigated between the two independent variables, daily use (yes/no) and QUEST score (1–5). The following model was investigated:

$$RR \sim QUES{T}_{score}+ {daily\ use}+ QUES{T}_{score}* {daily\ use}+ involvemen{t}_{quality}+ involvemen{t}_{duration} + trainin{g}_{intensty} + trainin{g}_{duration} + (1|discipline)$$

The different levels of each factor variable were always compared to the reference (and lowest) level, e.g., different training intensities were compared to the lowest possible selection of “2 h or less per week”. The linear mixed effects model was fitted in R using the *lmerTest* package.

## Results

In total, 81 responses from 35 PIL and 46 TL were collected. All teams partaking in the included disciplines of the CYBATHLON 2020 Global Edition were contacted, and 81.8% (36 of 44) were able to participate in our survey despite the stressful time days before the competition. The residual eight teams from the competition were unable to participate in favor of focusing on their last preparations and training before the races. Additionally, five teams that had to forfeit participation in the last couple of days before the event were also included in this study. Therefore, responses from a total of 41 teams were included. For some of the teams, either only the PIL or the TL completed the survey, while in others, more than one PIL and/or TL were included. Completed information (min. 1 PIL and TL) was obtained from 32 teams. The participant demographics are detailed in Table 1.

**Table 1:** Respondent demographics

	Pilot (n = 35)		Technical (n = 46)		Total (n = 81)	
	Number	Percentage	Number	Percentage	Number	Percentage
Gender						
Female	3	8.6%	8	17.4%	11	13.6%
Male	32	91.4%	38	82.6%	70	86.4%
Age						
18–24	1	2.9%	1	2.2%	2	2.5%
25–34	13	37.1%	29	63.0%	42	51.9%
35–44	9	25.7%	10	21.7%	19	23.5%
45–54	9	25.7%	4	8.7%	13	16.0%
55–64	3	8.6%	2	4.3%	5	6.2%
CYBATHLON discipline						
Powered arm prosthesis race	9	25.7%	15	32.6%	24	29.6%
FES bike race	8	22.9%	10	21.7%	18	22.2%
Powered exoskeleton race	8	22.9%	10	21.7%	18	22.2%
Powered leg prosthesis race	5	14.3%	6	13.0%	11	13.6%
Powered wheelchair race	5	14.3%	5	10.9%	10	12.3%
Home continent						
Europe	21	60.0%	27	58.7%	48	59.3%
Asia	9	25.7%	12	26.1%	21	25.9%
North America	3	8.6%	5	10.9%	8	9.9%
Africa	1	2.9%	1	2.2%	2	2.5%
Latin America	1	2.9%	1	2.2%	2	2.5%
CYBATHLON 2020 participant						
Yes	31	88.6%	40	87.0%	71	87.7%
No	4	11.4%	6	13.0%	10	12.3%

### User involvement during device development

**Fig. 1:**
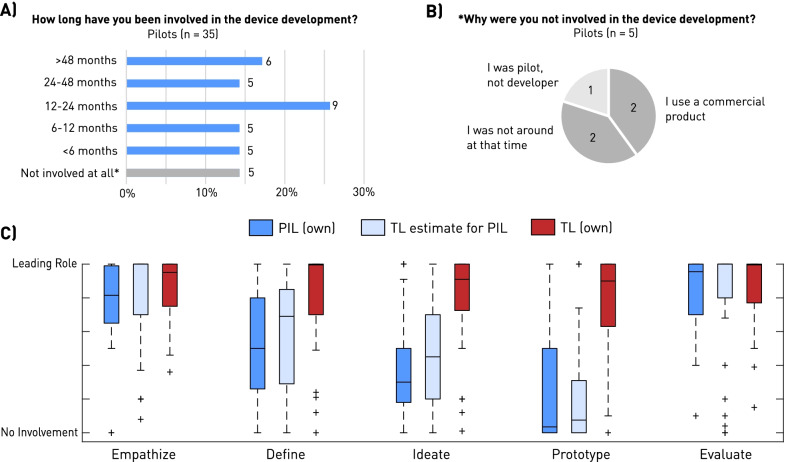
Device development involvement of pilots and technical leads: **A** Duration/quantity of PIL involvement (n = 35), **B** Reasons for no PIL involvement during device development (n = 5), **C** Quality of development involvement, ranging from leading role (100%) to no involvement (0%). PIL only estimated their own involvement (dark blue), while TL estimated the PIL involvement (light blue), and their own (red)

Figure 1A shows the duration (in months) of PIL involvement in the device development. Only 5 out of 35 PIL reported no involvement in the development, indicating that 85.7% of all PIL considered themselves being an active member of the development team. In terms of involvement duration, 28.5% of PIL appear to be involved for 12 months or less, and 17.1% for more than 4 years. No average involvement duration was calculated as the responses were categorical. Reasons for no involvement appeared to be depending on device maturity, definition or understanding of team role, as well as personal availability (Fig. 1B). Figure 1C depicts the involvement quality of the two respondent groups. Compared to the PIL, the TL are in a leading role throughout the development cycle, i.e., during all individual development phases. In contrast, PIL are mainly highly involved during problem characterization (*empathize, define*) and the eventual assessment of solutions (*evaluation*), but rarely in the “engineering” phases of ideation and prototyping. Outliers in TL data were identified as project- or team managers, acting in an organizing and facilitating role rather than leading the device development from the technical perspective. From the perspective of the TL, all 46 respondents (100%) indicated that the PIL were involved in the development. No significant difference between the estimations of PIL involvement from the TL perspective (Fig. 1C, light blue), and the PIL own estimates was observed. The matching estimates indicated by the comparable lines connecting the mean involvement estimates validate the overall impression on PIL involvement quality.

**Fig. 2:**
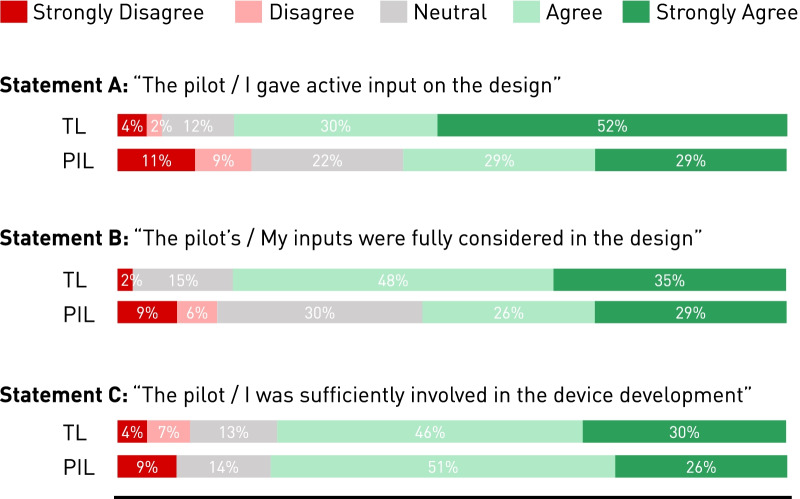
Reflection on pilot involvement: Level of agreement from technical leads (TL, n = 46) and pilots (PIL, n = 35) with three statements A, B and C on the PIL involvement during device development. Agreement is indicated in green, neutral opinions in grey, and disagreement in red. The full bar represents 100%, with levels of disagreement and agreement stacked visually

Additional insights on the quality of PIL involvement were generated through the quantified level of agreement with statements A, B, and C (Fig. 2). For all three statements, more than 50% the PIL and TL stated a certain level of agreement. However, a difference in opinion between the TL and PIL for statements A and B could be observed, with PIL showing a larger share of disagreement and neutral sentiments. While, the TL appear to judge the PIL involvement as very active and their needs being fully considered (82% and 83% agreement, respectively), the PIL showed a substantial amount of disagreement with statements A and B (20% and 15%, respectively). A neutral opinion on the three statements could be considered somewhat negative, further increasing the observed difference of opinion between TL and PIL. Common levels of agreement between the PIL and TL was found on the quantitative adequacy of pilot involvement (Fig. 2, Statement C).

### Usability of CYBATHLON devices

**Fig. 3:**
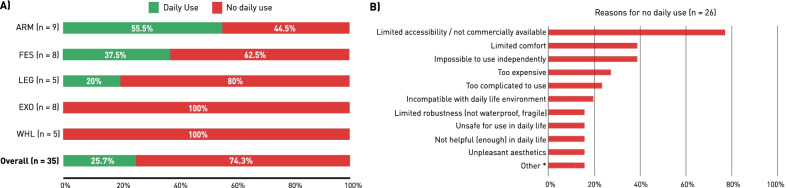
Daily life usage of CYBATHLON-specific AAT: **A** PIL indications of whether or not they use the showcased AAT in daily life, outside of CYBATHLON-related activities (n = 35), **B** List and frequency of reasons preventing daily use, as stated by PIL that indicated no daily use of their CYBATHLON-specific AAT (n = 26), *Other (paraphrased from translations) = “*limited reliability”, “low-tech missing sensation”, “FES tech limitation”*, “*it’s a prototype that needs refinement”, “underdeveloped legislation and subsidization for purchase, maintenance, and warranty insurance, “limited battery life”*

Figure 3A shows that of all 35 PIL interviewed, only 25.7% use their CYBATHLON-specific device in daily life. Devices of the ARM discipline (upper limb prostheses) reported the highest daily usage outside of CYBATHLON-related activities, with 5 out of 9 PIL using their device in daily life (55.5%). With 37.5%, FES devices where the second most used AAT type in daily life. Their usage form was mostly reported as stationary exercise at home. None of the devices in the WHL and EXO disciplines were reported to be used outside of controlled environments such as training for the CYBATHLON. The main preventive factors of daily life usage were reported to be the limited accessibility, non-availability, or excessive costs of the AAT developed for the CYBATHLON (Fig. 3B). These factors can therefore be considered the main technology adoption barriers. In terms of usability (during use), insufficient comfort, as well as the complicated or help-dependent use were mostly mentioned as current limitations.

**Fig. 4:**
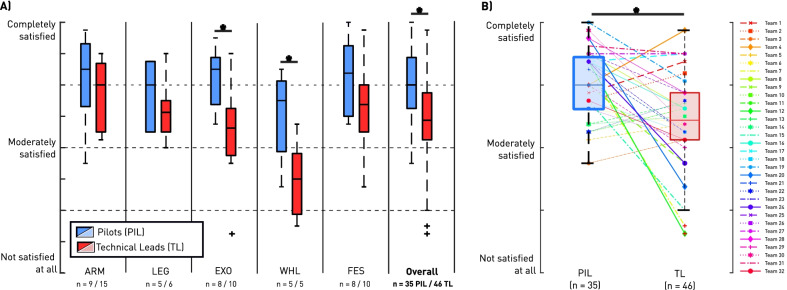
QUEST 2.0 subjective ratings of AAT usability: **A** The mean QUEST ratings of both respondent groups (PIL; n = 35, TL; n = 46) are shown per discipline, as well as overall (mean across all disciplines). The QUEST rating scale ranges from 0 = *Not satisfied at all*, to 5 = *Completely satisfied*. Significant differences were found for the EXO (p = 0.014) and WHL (p = 0.049) disciplines, as well as the overall mean (p = 0.019). **B** The QUEST mean of each individual response is shown for both respondent groups. For all teams with at least one PIL and TL response, an intra-team line is drawn to highlight differences of opinion about the same AAT device (p = 0.019). For all teams with multiple responses (e.g., two TL for team), the overall mean per subgroup is shown

Considering the observed minimal daily use, as well as the limitations listed, we expected the QUEST 2.0 ratings to reflect a certain dissatisfaction of the PIL regarding their device, shown in Fig. 4. Surprisingly, the mean QUEST scores of the PIL across all disciplines reported a moderate to high satisfaction, with an overall mean of 3.98 from a maximum of 5.0 = *Complete satisfaction* (Fig. 4A). Significantly lower ratings by the TL were observed for the EXO (p = 0.014) and WHL (p = 0.049) disciplines when compared to the discipline mean across all PIL. The overall means (mean of all disciplines) of the two respondent groups differed significantly (p = 0.019), with the TL rating the device usability lower on average. In Fig. 4B, individual responses by the PIL and TL are plotted, and the intra-team connections indicated. This visualization allowed to observe that despite the fact that the overall mean of the TL was significantly lower, not all TL rated the QUEST 2.0 lower than their team’s PIL. Further, we can observe that only for very few teams, the intra-team lines are horizontal, which would indicate a similar rating of the same device’s usability. Even more so, the lines for individual teams, such as for Team 12 and 20, are very steep, indicating substantial differences in opinions, or perceptions between team members.

### Competition performance indicators

**Fig. 5:**
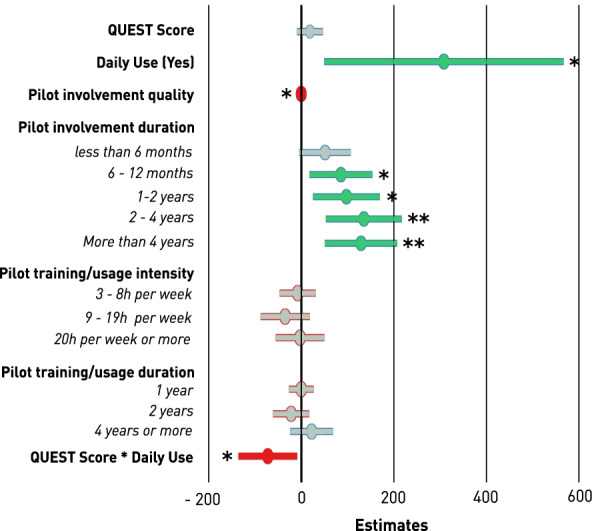
Effect estimations of user-centered design variables on CYBATHLON performance: The individual estimates of each independent variable from the explorative mixed linear model are visualized. Variables and factor levels indicated in green showed a significant positive effect on RR (higher score/category = higher RR) and those indicated in red showed a significant negative effect (higher score/category = lower RR). Variables in green did not show a significant effect. Levels of significance are remarked as * = p < 0.05, ** = p < 0.01 and *** = p < 0.001

From all PIL responses collected, 31 could eventually be linked to their team’s performance (RR) at the CYBATHLON 2020 Global Edition. A mixed linear model to explore the fixed effects of independent variables describing PIL involvement, usage intensity as well as usability on the dependent variable RR was fitted. The model summary with all estimates and individual p-values can be found in the Additional file 1. A forest plot indicating the positive and negative effects of each independent variable, in form of their estimates and 95% confidence intervals, is visualized in Fig. 5. The most significant positive effect on the RR, and thus the most relevant indicator for a good performance at the CYBATHLON competition, is the daily use of the AAT by the PIL (p = 0.035). Involvement duration also positively affects the performance, with an increase of the estimated effect observable until a PIL involvement duration of 2–4 years. In the explorative model, CYBATHLON-specific training intensity (hours of training per week) does not appear to affect the RR significantly, whereas a higher training has a positive influence on the competition performance after more than 4 years of training. The interaction effect between the QUEST usability rating and the daily use variable was found to be negatively affecting the performance (p = 0.042).

## Discussion

In an online survey for the PIL and TL of teams preparing for the CYBATHLON 2020 Global Edition, we collected data from the unique pool of developers and users of specialized AAT, with the objective of shedding light on the influence of a competition like CYBATHLON on the development and acceptance of AAT. More specifically, we analyzed the PIL role during device development, the actual daily usage and usability ratings of the devices, as well as the effects of those user-centered design characteristics on the individual CYBATHLON race performances.

### Pilot involvement in CYBATHLON teams

One of the main results of this study was to confirm that across all investigated disciplines, more than 85% of the PIL played a vital role in large parts of the device development. From the 35 PIL surveyed, 57% have been involved in their team’s endeavors for more than 1 year. This somewhat implies that for most of the teams, the PIL role is not limited to testing and training the finalized device to compete at the event but rather to be an integral part of the team during development. Still, when looking at the individual UCD phases, we can understand that—not surprisingly—the pilots are primarily involved in empathizing and defining the problem or goal to be addressed and when evaluating the technical solution (mostly referred as “training”). Especially in the last weeks before the competition, the PIL involvement becomes more intensive, with multiple PIL reporting more than 20 h of training per week. An increase in training intensity up to daily practice in the last days before the competition was previously reported by several teams participating in the 2016 competition [19, 23]. Other experiences, such as reported from the VUB-CYBERLEGS team in 2016, show that as little training as a total of 14 h with the device can suffice for the PIL to participate in the competition successfully [21]. Intensive involvement of the PIL in any CYBATHLON-related activities might be expected, but surely cannot be taken for granted. From anecdotal evidence shared in the past, we understand that in a majority of the participating teams, the PIL contribute on voluntary and non-paid basis, while following regular day-to-day lives and jobs. In the meantime, most technical leads (students, researchers, staff, industrial engineers, etc.) dedicate their daily work to device development. Reasons for the minor, or lacking PIL involvement in the technical development could therefore be: (1) limited resources, i.e., time and budget, (2) limited technical expertise of PIL (3) limited motivation or need. A reduced involvement of the PIL in the more technical phases of device development might be a reason for partial disagreement of the PIL’s personal impact and consideration in the final design, as observed in the Likert Scale answers. Only in a few teams, the PIL simultaneously are among the leading engineers, or researchers behind the technical solutions to implement their needs and wishes directly [32, 33]. The employment status and actual workload of PIL within development teams remain somewhat undocumented, restricting a more detailed analysis.

In contrast to the PIL responses, all TL indicated that the PIL were involved in the device development. This mismatch could be due to some PIL interpreting the wording “*involved during device development*” more critically, as training and using the device for the competition could be arguably seen as involvement per se. Based on the results of similar works such as by Ármannsdóttir et al., in which developers of powered lower-limb exoskeletons for therapy and daily assistance reported a target user involvement rate of 97%, a certain level of involvement from all PIL seems likely. Therefore, the main question is not if, but in which development phases, and how users play an active part of the development team. In the majority of AAT developments, target users only get involved once the engineering teams have spent substantial resources developing and testing with neurologically intact, mock-users [33]. At this point, there is a high probability that the technical solutions have slightly diverted and evolved away from the target-user needs and problems to be addressed. This often results in an unsatisfactory usability from the perspectives of target users. It appears that for the teams participating at the CYBATHLON, the pilot-centered competition concept may actively help tackle this issue, as many PIL get involved in the projects from early on, and throughout the device development. Involvement in technical development phases such as prototyping does not necessarily imply that the PIL have to program software or solder electronics themselves. User-centered design can be supported with various collaborative design methods such as focus groups, workshops, storyboards, or cognitive walkthroughs to establish or discuss concepts, ideas, and prototypes [34]. In any stage of development of (advanced) assistive technology, user feedback is essential to maintain a user-centered focus by following up on user ideas, needs, or emotions [9, 14]. The combined insights on the PIL and TL perceptions and experiences on their involvement in AAT development suggest that the quality and quantity of user involvement might still be improved with additional co-creation and usability methods or be enhanced with more resources to incorporate the PIL closer to the hands-on development. Still, we can conclude that UCD is common practice among the CYBATHLON teams, as it also had been reported in experience reports from teams of the 2016 competition [23, 30]. The CYBATHLON as a platform promotes the exchange and collaboration between people with disabilities and AAT developers, thereby reaching one of its seminal objectives [12, 13].

### Daily life applicability and usability

Strongly linked to user involvement, another main interest of this study was to investigate the daily life usage, and subjectively rated usability of the AAT developed explicitly for the CYBATHLON. From the mean QUEST scores, we understand that, surprisingly, the TL appeared to be less satisfied with their developed technology than the actual end-users, the PIL. Specifically, in the disciplines that ask for more complex technical solutions, namely, the EXO and WHL races, the TL see plenty of room for improvement. This is in contrast to the PIL perceptions, which indicate an encouraging level of satisfaction (QUEST mean = 3.98) with the AAT usability [30]. Given that the QUEST is one of the most used scales for the subjective rating of perceived usability [34, 36], the mean QUEST scores from our data might serve as an approximate benchmark for other AAT. However, the comparison to daily life usability ratings has to be interpreted cautiously as the CYBATHLON is a highly specific context of use. The reported actual daily use—as a consequence of successful technology acceptance and adoption—is arguably a more reliable daily life usability outcome of the AAT showcased at the CYBATHLON. With only 25% of all PIL using their AAT in everyday life, prevalent usability limitations and adoption barriers were observed. A large number of PIL reported limited availability as the main inhibiting factor, meaning that they do not have access to the technology in daily life. Additional adoption barriers such as too high costs or incompatibility with the daily environment were also prevalent. Such a limited availability and accessibility of AAT showcased at the CYBATHLON can be explained by the fact that most teams work with devices that are either exclusively built for the CYBATHLON, or existing research devices that are optimized for the specific race conditions. Only a minority of devices are commercially available and have regulatory approval to be used without technical supervision. Hardware and software functions are often optimized to overcome the exact obstacles or racetracks that are known to the participants months in advance. Usage of the devices outside of the controlled environment of a CYBATHLON obstacle course requires significantly more dynamic control, feature adaptability, and additional safety precautions. Still, given that the devices are often tailored to the individual PIL, it is somewhat surprising that only very few of them use their AAT in daily life, besides any activities related to the competition. It thus remains difficult to judge if the specific developments towards a CYBATHLON participation only positively influence the daily life usability of the AAT. In terms of usability limitations, insufficient comfort and overcomplicated use (requiring technical assistance) were identified factors inhibiting daily use. From the factors listed, PIL indicated that the effectiveness (functionality, reliability, helpfulness, etc.) of their AAT is not the leading limitation, but rather that usage efficiency and overall satisfaction need to be improved to motivate for or enable daily use. These insights align with recent reports on the current state of usability of wearable robotic technologies [18, 31]. It is to be expected that if such usability limitations cannot be addressed, target users will likely abandon their AAT soon after an eventual acquisition [2, 3].

### CYBATHLON race performance indicators

Lastly, we were interested to investigate the potential link between performance in the CYBATHLON and user-centered design variables like user involvement intensity and perceived usability. For this purpose, we fitted a mixed model of several UCD variables expected to have an effect on the individual race performances at the CYB. We analyzed to what extent user involvement, usage- and training intensity, and usability ratings could be defined as performance indicators. Daily AAT use beyond CYBATHLON-specific training was found to be most positively affecting race performance. PIL who can maneuver and control their device in daily life are thus likely to perform well at the CYBATHLON, indicating the racetracks’ relevances to activities of daily living. Also, we could identify that the duration of PIL involvement (involvement quantity) appears to be a main performance-indicator. The increasing, positive effect of long-term involvement, however, appears to reach a plateau with PIL contributions beyond 4 years. This does not indicate that involving a PIL for more than 4 years is not beneficial for a good performance at the CYBATHLON, but rather that there is no significant difference in the positive effect compared to involvements of 2–4 years duration. The re-occurring success stories from teams that participated in both competitions of 2016 and 2020 with the same PIL, like HSR enhanced (WHL), Cleveland (FES), TWIICE (EXO), or IHMC (EXO) confirm this identified benefit of long-term PIL involvement [23, 26, 37, 38]. Interestingly, the quality of PIL involvement, i.e., their role within the five different development phases, was not found to significantly affect race performance. According to our data, a training intensity higher than two hours per week, as well as prolonged training over more than six months do not appear to significantly improve the performances at the 2020 competition. From reports of participants in 2016, however, we can understand that prolonged training might positively affect performances based on observations of reduced mental, or physical load, as well as reduced task times in the progression of prolonged task-specific training [21, 23]. The satisfaction of the PIL regarding the usability of their AAT also does not appear to be a significant factor affecting their race performance, indicating that the ability and willingness of the PIL to adapt to the available functions (or limited usability) of the device might be more crucial within the competition. We can link this observation to the results from the ARM discipline in both the 2016 and 2020 competition, where more simple technologies with limited number of features, in these cases body-powered prostheses, appeared to perform better. This may be due to the fact, that the PIL have to control less parameters, inevitably making simple systems easier to use in demanding, or stressful situations [39]. If such simplified systems also yield a high usability in daily life is yet to be investigated. A solid interpretation of the negative estimate that was linked to the interaction effect is rather difficult, as the two variables appeared to be more collinear than interactive. Overall, it is important to state that the performance of the teams at the CYBATHLON is not only influenced by the few UCD variables discussed in this work. Innovative technological solutions and competition tactics to complete the CYBATHLON tasks more efficiently, or effectively can potentially bring a large advantage to the PIL [31]. Still, it is the PIL and their expertise in handling the AAT under the pressure of a competition format, which decides what they can make out of the few chances during race qualifications and finals. Our explorative analysis solely aimed to investigate if and how UCD can help achieving a better performance at the CYBATHLON, and thus imply that it is likely to improve usability and technology acceptance of AAT.

### Limitations

As for most usability studies that use co-creators as subjects, our survey results are likely influenced by certain cognitive biases (e.g., recency bias, social desirability bias, etc.), limiting the respondent’s fair and critical judgment [40]. Also, survey results are inherently subject to response biases such as recency-, recall-, or acquiescence bias. The cross-sectional design of the study limits the analysis of actual effects of PIL involvement in individual teams and projects. A longitudinal assessment would be needed, tracking the team's development and the user sentiment before, during, and after a CYBATHLON competition. This survey therefore only represents a starting point of user-centered design data collection at and around the CYBATHLON platform. Another limitation worth mentioning was the multi-lingual approach that was taken. Even though professionals and subject matter experts were involved in the translations, the fact that the surveys were offered and collected in nine different languages might have led to minor misunderstandings or misinterpretations. However, the benefit of increasing sample numbers likely overweighs those limitations, also because numerous respondents appreciated and complimented the comprehensible translations. The still relatively small and varying sample sizes between disciplines can be listed as a factor limiting certain conclusions drawn from this study. Moreover, the sample group of TL did not solely consist of lead engineers but also of project managers, application specialists or other team members. The interpretations from the linear mixed model should be understood as an explorative analysis, as the user-centered design variables used for the model differed in factor levels and variable types and showed certain collinearities that make a clear statistical distinction of their effects very difficult. Also, the list of variables that influence the CYBATHLON performance is clearly more extensive, with many psychological (stress, cognitive load, etc.), technical (team tactics, device malfunctions, device complexities, etc.), and economic factors (team sizes, available resources, etc.) not being addressed within this explorative analysis.

### Implications and importance of the study

Our survey results show that the CYBATHLON appears to achieve its conceptual goals of encouraging user-centered design among the participating teams by promoting inclusion and exchange between AAT stakeholders. While CYBATHLON successfully stimulates new developments of specialized, high-performance AAT, the technological transfer and translation to devices that can be used in daily life outside of the competition remain a challenge. The limited daily use of the CYBATHLON-specific AAT also raises the question of whether developments specifically directed towards the races, and their clearly defined tasks, may somewhat be counterproductive to the generalization and robustness of the showcased functionalities for the more dynamic, unstructured environments in daily life. Furthermore, the pilots could arguably be seen as a rather technology savvy courageous subgroup of their respective AAT target population. Their dedication and willingness to train towards the CYBATHLON competitions might not reflect the average effort another user would face when acquiring such AAT for use in daily life. The intensive, goal-oriented design approach involving the pilot in the AAT development might thus be feasible and essential in the context of participation to CYBATHLON, but its effects on race performance and usability might not be directly applicable in a more general context. It thus remains difficult to assess the extent to which the CYBATHLON influences the general AAT development and market. The competition and its outreach are attractive for sponsors, granting projects funding for developments towards the competition. After the competition, that funding alone is likely insufficient to allow for further developments to push towards commercialization of promising devices, or individual features. Besides monetary resources, it is crucial that conditions prevail which favor the transformation of prototypes into market-ready products such as structured knowledge transfers, reimbursement strategies, or AT market readiness. Besides individual projects that directly benefited from their CYBATHLON participation to eventually incorporate and commercialize their technology, like TWIICE [41], Caterwil [42], or Scewo [43], we understand that the majority of AAT showcased at the competitions might not become commercially available per se. However, the competitive spirit and challenges posed at the CYBATHLON are expected to bring forward technical innovations, social interactions, and manifold learnings that positively influence the overall technology readiness and transfer of novel AAT.

### Recommendation for future research and applications

Based on our interactions with the teams of the CYBATHLON 2020 Global Edition competition, we could gather and analyze valuable data to investigate user involvement strategies, usability hurdles, and performance-indicating factors and further get an in-depth understanding of how the platform promotes user-centered design on a general level. The PIL are, one way or another, an active part of the development teams. We learned that the quality and quantity of this user involvement may differ among the teams and that a majority of PIL appeared to be only minorly involved in the technical development phases. Despite potentially limited resources, we would recommend to promote AAT target user involvement in the phases of conceptualization and prototyping in order to maintain focus on their needs. Further, we could indicate that PIL are likely to perform better at the competitions if they spend as much time with the devices as possible. Optimally, PIL should be able to use their AAT in daily life and therefore have access to it outside of the laboratory environment where training typically takes place. In the near future, we hope to see the number of PIL using their CYBATHLON-specific AAT in daily life to grow. For this to happen, both the teams and the competition rules have to make sure to maintain focus on solving actual daily life problems of people with disabilities. As mentioned above, this survey now marks a starting point of further, longitudinal assessments at, and around the CYBATHLON competitions. Eventually, we might be able to track which innovations translate to industry and how the CYBATHLON impacts the AAT in the long-term perspective. The insights generated by this survey are further transferrable to CYBATHLON-unrelated AAT developments, highlighting the importance of a user-centered mindset and development strategy.

## Conclusions

Since its initiation in 2016, the CYBATHLON has become a leading platform to promote the usage and design of novel, robotic technologies in close collaboration with people with disabilities. Although the competitional format may favor the development of highly-specialized technologies, the platform offers a unique display and reflection of the current technology maturity of AAT, with remaining usability limitations and technology adaption hurdles being revealed. Particularly for younger, emerging technologies like powered exoskeletons and advanced powered wheelchairs, the format is likely to promote new, more robust, and more reliable solutions. However, the technological transfer of these solutions into daily life applications is not yet clear and will have to be monitored in the future. We believe that the CYBATHLON will continue to positively affect the AAT field, mainly through its core value of promoting the inclusion and empowerment of target users with disabilities in development teams and the general society.

## Supplementary Information

**Additional file 1.** **Supplementary Material 1.** Full survey: QuestionPro export (PIL and TL survey trees). **Supplementary Material 2.** Summary (readout) of linear mixed model.

## Data Availability

Data and materials can be made available upon request to the authors.

## Abbreviations

AAT: Advanced assistive technologies
UCD: User-centered design
TL: Technical lead
PIL: Pilot
ARM: Powered arm prosthesis race
LEG: Powered leg prosthesis race
EXO: Powered exoskeleton race
WHL: Powered wheelchair race
FES: Functional electrical stimulation bike race
BCI: Brain-computer interface race
QUEST: Quebec User Evaluation of Satisfaction with Assistive Technology
RR: Relative rank
