# Supplementary material for: A survey on the influence of CYBATHLON on the development and acceptance of advanced assistive technologies
Source: J Neuroeng Rehabil. 2022 Apr 2;19:38. doi: 10.1186/s12984-022-01015-5 (PMC8976279; doi:10.1186/s12984-022-01015-5)
Supplement: Supplementary file 1 — Additional file 1. Supplementary Material 1. Full survey: QuestionPro export (PIL and TL survey trees). Supplementary Material 2. Summary (readout) of linear mixed model. [file 12984_2022_1015_MOESM1_ESM.pdf]

A survey on the influence of CYBATHLON on the development and acceptance of advanced assistive technologies

Jan T. Meyer[1*], Selina Weber[1], Lukas Jäger[2], Roland Sigrist[2], Roger Gassert[1,2,3], Olivier Lambercy[1,3]

* Correspondence: publications@relab.ethz.ch

[1] Rehabilitation Engineering Laboratory, ETH Zürich, Zürich, Switzerland

[2] CYBATHLON, ETH Zürich, Zürich, Switzerland

[3] Future Health Technologies, Singapore-ETH Centre, Campus for Research Excellence And Technological Enterprise (CREATE), Singapore

Supplementary Material 1 (pages 2-14) – Full survey: QuestionPro export (PIL and TL survey trees)
Supplementary Material 2 (page 15) – Summary (readout) of linear mixed model

Supplementary Material 1 – Full survey: QuestionPro export (PIL and TL survey trees)

CYBATHLON 2020 Survey Introduction Text

Thank you for participating in our CYBATHLON 2020 survey. Your answers will help us understand to what extent users are involved in the development process of assistive technologies to identify which methods are used to promote user-centered design and assess device usability to evaluate the impact of the CYBATHLON on user involvement and device usability tp determine how close the developed technologies are to application in daily life. We kindly ask you to answer the questions below, according to your personal opinion. To fully complete the survey, approximately 15-20 minutes are required. If you need further information to understand and answer a question, please click on the help sign (if provided). For all additional questions, do not hesitate to consult the survey coordinator. This work is supported by the Swiss National Science Foundation through the National Centre of Competence in Research on Robotics and the Vontobel Foundation. The data will be stored anonymously on the secured servers of QuestionPro (temporary) and ETH Zurich (permanently) with access limited to the survey coordinators. The anonymized data may be shared and made public.
You may refuse to participate without consequences. If you decide to participate, you are free to quit the survey at any time without consequences, by just stopping and/or telling the survey coordinator. By clicking "Next" below, you agree to these terms and conditions.

Please type in your response-ID, which you received from the study coordinator (Sample ID: cya9):

Pilot survey tree

What is your age?
1. 18-24
2. 25-34
3. 35-44
4. 45-54
5. 55-64
6. Above 64

What is your gender?
1. Male
2. Female
3. Non-binary
4. Prefer not to disclose
5. Prefer to self-describe __________

What is the highest level of education you have completed?
1. Compulsory/elementary school qualifications
2. High school diploma (or equivalent)
3. Apprenticeship/technical training
4. Bachelor's degree
5. Master's degree
6. Doctoral degree
7. Other __________

Do you have a technical/engineering background?

1. Yes
2. No

In which CYBATHLON discipline do you compete with your device?

1. Powered Arm Prosthesis Race
2. Powered Leg Prosthesis Race
3. Powered Exoskeleton Race
4. Powered Wheelchair Race
5. Functional Electrical Stimulation (FES) Bike Race

The device I am using at the CYBATHLON 2020...

1. ... was built from scratch (for me)
2. ... builds on an existing/previous (research) prototype
3. ... is a (modified) commercial product
4. Other __________

Have you been personally involved in the device development? If yes, for how long?

1. No, not at all
2. Less than 6 months
3. 6-12 months
4. 1-2 years
5. 2-4 years
6. More than 4 years

To what extent were you personally involved in the following device development stages? 0 = not involved at all, 100 = leading role

Empathizing with the user needs	❑
Defining device requirements	❑
Formulating design ideas and concepts	❑
Creating prototypes/devices	❑
Testing and evaluating prototypes/devices	❑

Why were you not involved in the device development?You can choose multiple options and/or add others

1. I was not asked to
2. I use a commercial product
3. I was not around at that time
4. I did not want to
5. Other __________

To what extent do you agree with the following statements about your development involvement?

	Strongly disagree	Disagree	Neutral	Agree	Strongly agree
I gave active input on the design	❑	❑	❑	❑	❑
My inputs were fully considered in the design	❑	❑	❑	❑	❑
I was sufficiently involved in the device development	❑	❑	❑	❑	❑

To what extent is an improvement in performance with your device depending on training, and/or on design optimizations?

	Completely depending on design optimizations	Rather depending on design optimizations	Equally depending on both	Rather depending on training	Completely depending on training
In your current scenario, given your resources (materials, time, costs, etc.)	❑	❑	❑	❑	❑
In an ideal scenario, given unlimited resources (materials, time, costs, etc.)	❑	❑	❑	❑	❑

Please rank the following attributes of your device, according to their importance for daily use:1 = most important, 4 = least important

- Satisfaction(comfort, adaptability, pleasure, etc.) ___________
- Effectiveness(functionality, reliability, accuracy, etc.) ___________
- Efficiency (time, mental workload, physical workload, etc.) ___________
- Safety(ergonomics, risk-free use, etc.) ___________

How satisfied are you with...

	Not satisfied at all	Not very satisfied	More or less satisfied	Quite satisfied	Very satisfied
... the dimensions (size, height, length, width) of your device?	❑	❑	❑	❑	❑
... the weight of your device?	❑	❑	❑	❑	❑
... the ease in adjusting (fixing, fastening) the parts of your device?	❑	❑	❑	❑	❑
... how safe and secure your device is?	❑	❑	❑	❑	❑
... the durability (endurance, resistance to wear) of your device?	❑	❑	❑	❑	❑

... how easy to use your device is?	❑	❑	❑	❑	❑
... how comfortable your device is?	❑	❑	❑	❑	❑
... how effective your device is (i.e. the degree to which your device meets your needs)?	❑	❑	❑	❑	❑

How do you currently control the device's actions to complete the obstacle/race course?You can choose multiple options. If you are unsure, please specify your input method in "Other"

1. Conventional direct control inputs (e.g. manual buttons, switches, touch displays, joysticks)
2. Non-conventional direct control inputs (e.g. voice control, sip-and-puff, tongue switches)
3. Muscle signals (non-invasive, e.g. EMG, MMG)
4. Brain signals (non-invasive, e.g. EEG, fNIRS)
5. Other ___________

If you could choose freely, how would you like to control the device's actions?You can choose multiple options. If you are unsure, please specify your input method in "Other"

1. Conventional direct control inputs (e.g. manual buttons, switches, touch displays, joysticks)
2. Non-conventional direct control inputs (e.g. voice control, sip-and-puff, tongue switches)
3. Muscle signals (non-invasive, e.g. EMG, MMG)
4. Brain signals (non-invasive, e.g. EEG, fNIRS)
5. Other ___________

To what extent do you agree with the following statements?

	Strongly disagree	Disagree	Neutral	Agree	Strongly agree
The device always follows my intentions	❑	❑	❑	❑	❑
I had to learn a lot before I was confident controlling the device	❑	❑	❑	❑	❑
Controlling the device is very mentally demanding	❑	❑	❑	❑	❑
I would accept the risk of a surgery (e.g. implanting electrodes, transfer of nerve endings) in exchange for a more natural control of my device	❑	❑	❑	❑	❑

For how long have you been testing/training on the CYBATHLON obstacle/race course?

	Less than 6 months	1 year	2 years	3 years	4 years or more
Please place the slider	❑	❑	❑	❑	❑

In the last two months, how many hours per week (on average) did you spend training and practicing with the device?

	Less than 1 hour	2 hours	4 hours	6 hours	8 hours	10 hours	12 hours	14 hours	16 hours	18 hours	20 hours or more
Please place the slider	☐	☐	☐	☐	☐	☐	☐	☐	☐	☐	☐

To what extent do you agree with the following statements about your discipline's CYBATHLON obstacle/race track?

	Strongly disagree	Disagree	Neutral	Agree	Strongly agree
The obstacle/race track represents realistic tasks of daily living	☐	☐	☐	☐	☐
Performing well at the race indicates high usability for daily life	☐	☐	☐	☐	☐
I am willing to take risks in order to win the race	☐	☐	☐	☐	☐
I think I have a good chance to win the race	☐	☐	☐	☐	☐

To what extent do you agree with the following statements about the CYBATHLON's influence on your life?

	Strongly Disagree	Disagree	Neutral	Agree	Strongly Agree
I feel more comfortable expressing my needs as an assistive technology user	☐	☐	☐	☐	☐
I feel representative of the target group of the assistive device I am using at the CYBATHLON	☐	☐	☐	☐	☐
The CYBATHLON has a positive effect on the social awareness and inclusion of people with disabilities	☐	☐	☐	☐	☐

Please rank the following statements about your personal goals and reward for your engagement at the CYBATHLON:1 = most important, 5 = least important

- I want to help with the development of novel assistive technology ___________
- I see myself as an athlete, competing at an international contest ___________
- I see the device usage as physical and mental training ___________
- My engagement is motivated by monetary compensation ___________
- I want to raise awareness for people with disabilities ___________

Are you using, or have you used the device in your daily life (besides CYBATHLON related

training/testing)?

1. Yes
2. No

How long have you been using your device in daily life?

	Less than 6 months	1 year	2 years	3 years	4 years or more
Please place the slider	❑	❑	❑	❑	❑

In which activities of daily living are you assisted/supported by your device?You can choose multiple options and/or add others

1. Work
2. Therapy
3. Leisure (sports, exercise, etc.)
4. Dressing, undressing
5. Mobility, transferring
6. Personal hygiene (washing, grooming, etc.)
7. Maintaining continence
8. Food preparation, eating, drinking
9. Other ___________

In which activities of daily living would you like to be assisted/supported by your device? You can choose multiple options and/or add others

1. Work
2. Therapy
3. Leisure (sports, exercise, etc.)
4. Dressing, undressing
5. Mobility, transferring
6. Personal hygiene (washing, grooming, etc.)
7. Maintaining continence
8. Food preparation, eating, drinking
9. Other ___________
10. None of the above

What are currently the most limiting factors restricting the daily use of your device? You can choose multiple options and/or add others

1. Too complicated to use
2. I cannot use it independently
3. Limited comfort
4. Unpleasant aesthetics
5. Unreliable functionality
6. Not helpful in daily life
7. Not compatible with daily life (home/work environment)
8. Not safe for use in daily life
9. Not robust (e.g. fragile parts, not waterproof, etc.)
10. Limited accessibility OR not commercially available yet
11. Too expensive
12. Other ___________
13. There is no limitation

Technical Lead survey tree

What is your age?
1. Under 18
2. 18-24
3. 25-34
4. 35-44
5. 45-54
6. 55-64
7. Above 64

What is your gender?
1. Male
2. Female
3. Non-binary
4. Prefer not to disclose
5. Prefer to self-describe __________

What is your professional/educational background?
1. Mechanical engineering
2. Electrical engineering
3. Computer sciences
4. Biomedical engineering
5. Physical therapy
6. Occupational therapy
7. Medicine
8. Health and/or movement sciences
9. Industrial design
10. Other __________

What is the highest level of education you have completed?
1. Compulsory/elementary school qualifications
2. High school diploma (or equivalent)
3. Apprenticeship/technical training
4. Bachelor's degree
5. Master's degree
6. Doctoral degree
7. Other __________

In which CYBATHLON discipline do you compete with your device?
1. Powered Arm Prosthesis Race
2. Powered Leg Prosthesis Race
3. Powered Exoskeleton Race
4. Powered Wheelchair Race
5. Functional Electrical Stimulation (FES) Bike Race

Did your team (university, company or else) already participate in the CYBATHLON 2016?
1. Yes
2. No

To what extent does your current device compare to the one your previous team used in 2016?
1. We use exactly the same device
2. We made minor changes on the previous device
3. We made major changes on the previous device
4. We use a completely different device
5. Other __________

The device we are using at the CYBATHLON 2020...
1. ... was built from scratch
2. ... builds on an existing/previous (research) prototype
3. ... is a (modified) commercial product
4. Other __________

What happens to the device after the CYBATHLON 2020?You can choose multiple options and/or add others
1. We give the device (back) to the pilot to use it in daily life
2. We will continue the device development without the current pilot(s)
3. We will continue the device development with the current pilot(s)
4. We aim to bring the device concept to the market
5. Other __________
6. I don't know OR no plans yet

For how long have you been personally involved in the device development?
1. Less than 6 months
2. 6-12 months
3. 1-2 years
4. 2-4 years
5. More than 4 years

To what extent were you personally involved in the following device development stages?0 = not involved at all, 100 = leading role

Empathizing with the user needs	❑
Defining device requirements	❑
Formulating design ideas and concepts	❑
Creating prototypes/devices	❑
Testing and evaluating prototypes/devices	❑

To what extent was your main pilot involved in the following device development stages?0 = not involved at all, 100 = leading role

Empathizing with the user needs	❑
Defining device requirements	❑
Formulating design ideas and concepts	❑
Creating prototypes/devices	❑
Testing and evaluating prototypes/devices	❑

To what extent do you agree with the following statements about the development involvement?

	Strongly disagree	Disagree	Neutral	Agree	Strongly agree
The pilot(s) gave active input on the design	❑	❑	❑	❑	❑
The pilot(s)' inputs were fully considered in the design	❑	❑	❑	❑	❑
The pilot(s) was/were sufficiently involved in the device development	❑	❑	❑	❑	❑

To what extent is a pilot's improvement in performance with your device depending on training, and/or on design optimizations?

	Completely depending on design optimizations	Rather depending on design optimizations	Equally depending on both	Rather depending on training	Completely depending on training
In your current scenario, given your resources (materials, time, costs, etc.)	❑	❑	❑	❑	❑
In an ideal scenario, given unlimited resources (materials, time, costs, etc.)	❑	❑	❑	❑	❑

Please rank the following attributes of your device, according to their importance for daily use:1 = most important, 4 = least important

- Satisfaction(comfort, adaptability, pleasure, etc.) ___________
- Effectiveness(functionality, reliability, accuracy, etc.) ___________
- Efficiency(time, mental workload, physical workload, etc.) ___________
- Safety(ergonomics, risk-free use, etc.) ___________

How satisfied are you with...

	Not satisfied at	Not very satisfied	More or less	Quite satisfied	Very satisfied

	all		satisfied		
... the dimensions (size, height, length, width) of your device?	❑	❑	❑	❑	❑
... the weight of your device?	❑	❑	❑	❑	❑
... the ease in adjusting (fixing, fastening) the parts of your device?	❑	❑	❑	❑	❑
... how safe and secure your device is?	❑	❑	❑	❑	❑
... the durability (endurance, resistance to wear) of your device?	❑	❑	❑	❑	❑
... how easy to use your device is?	❑	❑	❑	❑	❑
... how comfortable your device is?	❑	❑	❑	❑	❑
... how effective your device is (i.e. the degree to which your device meets the user's needs)?	❑	❑	❑	❑	❑

What general evaluation methods did you use to assess the usability of your device?You can choose multiple options and/or add others

1. Interview, unstructured/oral feedback
2. Questionnaires, surveys, scales
3. Performance-related measures (task completion, functional tests, time for task etc.)
4. Qualitative observation of pilots (video analysis, eye-tracking, etc.)
5. Quantitative observation of pilots (motion capture, eye-tracking, etc.)
6. Thinking aloud
7. Expert evaluation
8. Model- and simulation-based approaches
9. Document-based approaches
10. Other ___________
11. None of the above

Please rank the following evaluation methods, according to their usefulness to assess the usability of your device:1 = most useful, 9 = least useful; select at least 7 choices to rank them

- Interview, unstructured/oral feedback ___________
- Questionnaires, surveys, scales ___________
- Performance-related measures (task completion, functional tests, time for task, etc.) ___________
- Qualitative observation of pilots (video analysis, eye-tracking, etc.) ___________
- Quantitative observation of pilots (motion capture, eye-tracking, etc.) ___________
- Thinking aloud ___________
- Expert evaluation ___________
- Model- and simulation-based approaches ___________
- Document-based approaches ___________

How many pilots did your team recruit and test with to participate in the CYBATHLON 2020?

1. 1
2. 2
3. 3
4. More than 3

For how long has your team (with AND without pilot(s)) been testing/training on the CYBATHLON obstacle/race course?

	Less than 6 months	1 year	2 years	3 years	4 years or more
Please place the slider	❑	❑	❑	❑	❑

In the last two months, how many hours per week (on average) did you spend testing and training with your device (with AND without pilot(s))?

	Less than 1 hour	2 hours	4 hours	6 hours	8 hours	10 hours	12 hours	14 hours	16 hours	18 hours	20 hours or more
Please place the slider	❑	❑	❑	❑	❑	❑	❑	❑	❑	❑	❑

To what extent do you agree with the following statements about your discipline's CYBATHLON obstacle/race track?

	Strongly disagree	Disagree	Neutral	Agree	Strongly agree
The obstacle/race track represents realistic tasks of daily living	❑	❑	❑	❑	❑
Performing well at the race indicates high usability for daily life	❑	❑	❑	❑	❑

How many obstacles of your discipline's race track do you aim to overcome at the CYBATHLON 2020?

	1 obstacle	2 obstacles	3 obstacles	4 obstacles	5 obstacles	(All) 6 obstacles
Please place the slider	❑	❑	❑	❑	❑	❑

How many percent of your discipline's race track do you aim to overcome at the CYBATHLON 2020?

	20 %(240m)	40%(480m)	60%(720m)	80%(960m)	100%(1200 m)
Please place the slider	❑	❑	❑	❑	❑

Why do you not aim to overcome all (obstacles of) the race track?You can choose multiple options and/or add others

1. Due to the time restriction
2. Due to safety reasons
3. Due to limited functionality of the device
4. For strategic reasons
5. Other ___________

To what extent do you agree with the following statements about the CYBATHLON's influence on device development?

	Strongly disagree	Disagree	Neutral	Agree	Strongly agree
The device's usability benefited from the (aim of) participation at the CYBATHLON	❑	❑	❑	❑	❑
The CYBATHLON's regulations and restrictions limited the development of the device	❑	❑	❑	❑	❑
The target-user involvement in the development process was increased due to the CYBATHLON	❑	❑	❑	❑	❑
The CYBATHLON motivated our team to face new challenges	❑	❑	❑	❑	❑
My understanding and empathy for people with disabilities has increased due to the CYBATHLON	❑	❑	❑	❑	❑

To what extent do you agree with the following statements about the CYBATHLON's impact on rehabilitation technology in general?

	Strongly disagree	Disagree	Neutral	Agree	Strongly agree
The general research interest has increased, since the initiation of the CYBATHLON	❑	❑	❑	❑	❑
The CYBATHLON enabled more/new funding for our device/project	❑	❑	❑	❑	❑
The CYBATHLON has a positive effect on the social acceptance of robotic assistive technology	❑	❑	❑	❑	❑
The CYBATHLON allows for valuable comparison to the state-of-the-art technology	❑	❑	❑	❑	❑
The CYBATHLON facilitates the translation from research prototypes to market products	❑	❑	❑	❑	❑

In which activities of daily living is/are your main pilot(s) assisted/supported by the device (besides CYBATHLON related training/testing)?You can choose multiple options and/or add others

1. Work
2. Therapy
3. Leisure (sports, exercise, etc.)
4. Dressing, undressing

5. Mobility, transferring
6. Personal hygiene (washing, grooming, etc.)
7. Maintaining continence
8. Food preparation, eating, drinking
9. Other __________
10. None of the above

In which activities of daily living do you think the pilot(s) could be assisted/supported by the device (additionally)? You can choose multiple options and/or add others

1. Work
2. Therapy
3. Leisure (sports, exercise, etc.)
4. Dressing, undressing
5. Mobility, transferring
6. Personal hygiene (washing, grooming, etc.)
7. Maintaining continence
8. Food preparation, eating, drinking
9. Other __________
10. Not intended for daily life

What are currently the most limiting factors restricting the daily use of your device?You can choose multiple options and/or add others

1. Too complicated to use
2. Pilots cannot use it independently
3. Limited comfort
4. Unpleasant aesthetics
5. Unreliable functionality
6. Not helpful in daily life
7. Not compatible with daily life (home/work environment)
8. Not safe for use in daily life
9. Not robust (e.g. fragile parts, not waterproof, etc.)
10. Limited accessibility OR not commercially available yet
11. Too expensive
12. Other __________
13. There is no limitation

Supplementary Material 2 – Summary (readout) of linear mixed model

summary(fit)
Linear mixed model fit by REML. t-tests use Satterthwaite's method ['lmerModLmerTest']

Formula: relative_rank ~ QUEST_score * daily_use + involvement_quality + involvement_duration + training_intensty + training_duration + (1|discipline)

REML criterion at convergence: 169.2

Scaled residuals:

Min	1Q	Median	3Q	Max
-1.08099	-0.51299	0.06473	0.40401	1.79786

Random effects:

Groups	Name	Variance	Std.Dev.
discipline	(Intercept)	76.48	8.745
Residual		581.15	24.107

Number of obs: 31, groups: discipline, 5

Fixed effects:

	**Estimate**	**Std. Error**	**df**	**t value**	**Pr(>|t|)**	
(Intercept)	-49.70257	56.62992	14.32796	-0.878	0.3946	
QUEST_score	18.65628	14.22189	13.79464	1.312	0.211	
daily_use_yes	308.04598	131.94457	14.23078	2.335	0.0347	*
involv_quality	-0.22778	0.08651	14.45538	-2.633	0.01927	*
involv_duration_less than 6 months	49.22009	28.40878	14.93191	1.733	0.10377	
involv_duration_6-12 months	83.42611	34.75423	13.92168	2.4	0.03093	*
involv_duration_1-2 years	95.427	36.81168	14.9968	2.592	0.02041	*
involv_duration_2-4 years	133.00393	41.7855	14.2454	3.183	0.00652	**
involv_duration_More than 4 years	126.79887	39.90219	14.95222	3.178	0.00626	**
raining_intensty_3 - 8 hours	-7.83198	19.74441	14.99294	-0.397	0.6972	
raining_intensty_9 - 19 hours	-34.98215	27.16067	14.94227	-1.288	0.21735	
raining_intensty_20 hours or more	-2.82004	26.84815	14.96437	-0.105	0.91774	
training_duration_1 year	-0.04928	13.45249	14.79828	-0.004	0.99713	
training_duration_2 years	-22.07843	19.90436	10.59931	-1.109	0.29187	
training_duration_4 years or more	22.20349	23.48456	14.55044	0.945	0.35987	
QUEST_score:daily_use_yes	-72.49108	32.41155	14.46335	-2.237	0.04154	*

Signif. codes: 0 ‘***’ 0.001 ‘**’ 0.01 ‘*’ 0.05 ‘.’ 0.1 ‘ ’ 1
